# Assessment of Environmental, Sociocultural, and Physiological Influences on Women’s Toileting Decisions and Behaviors Using “Where I Go”: Pilot Study of a Mobile App

**DOI:** 10.2196/56533

**Published:** 2025-02-12

**Authors:** Abigail R Smith, Elizabeth R Mueller, Cora E Lewis, Alayne Markland, Caroline Smerdon, Ariana L Smith, Siobhan Sutcliffe, Jean F Wyman, Lisa Kane Low, Janis M Miller, The Prevention of Lower Urinary Tract Symptoms (PLUS) Research Consortium

**Affiliations:** 1 Division of Biostatistics and Informatics, Department of Preventive Medicine, Feinberg School of Medicine, Northwestern University, 680 North Lake Shore Drive, Suite 1400, Chicago, IL, 60611, United States, 1 312-503-1060; 2 Departments of Urology & Obstetrics/Gynecology, Loyola University Medical Center, Loyola University Chicago, Chicago, IL, United States; 3 Department of Epidemiology, School of Public Health, University of Alabama at Birmingham, Birmingham, AL, United States; 4 Division of Gerontology, Geriatrics, and Palliative Care, Department of Medicine, School of Medicine, University of Alabama at Birmingham, Birmingham, AL, United States; 5 Geriatric Research, Education, and Clinical Center, Birmingham Veterans Affairs Health Care System, Birmingham, AL, United States; 6 Arbor Research Collaborative for Health, Ann Arbor, MI, United States; 7 Division of Urology, Department of Surgery, University of Pennsylvania Perelman School of Medicine, Philadelphia, PA, United States; 8 Division of Public Health Sciences, Department of Surgery, Washington University School of Medicine, St. Louis, MO, United States; 9 Department of Obstetrics and Gynecology, Washington University School of Medicine, St. Louis, MO, United States; 10 School of Nursing, University of Minnesota, Minneapolis, MN, United States; 11 Department of Health Behavior and Biological Sciences, School of Nursing, University of Michigan, Ann Arbor, MI, United States; 12 Department of Obstetrics & Gynecology, School of Medicine, University of Michigan, Ann Arbor, MI, United States; 13 See Acknowledgments

**Keywords:** ecological momentary assessment, time location factors, voiding diary, voiding behaviors, population studies, mobile application, app, bladder health, data collection tool, decision support

## Abstract

**Background:**

Little is known about women’s decisions around toileting for urination and how those decisions influence moment-to-moment behaviors to manage bladder needs. The new smartphone app “Where I Go” captures such nuanced and granular data in real-world environments.

**Objective:**

This study aims to describe participant engagement with “Where I Go”, variation in novel parameters collected, and readiness for the data collection tool’s use in population-based studies.

**Methods:**

“Where I Go” has three components: (1) real-time data, (2) short look-back periods (3‐4 h), and (3) event location (GPS recorded at each interaction). The sample size was 44 women. Recording of real-time toileting events and responding to look-back questions was measured over 2 days of data collection. The participant’s self-entered location descriptions and the automatic GPS recordings were compared.

**Results:**

A total of 44 women with an average age of 44 (range 21-85) years interacted with the app. Real-time reporting of at least 1 toileting event per day was high (38/44, 86%, on day 1 and 40/44, 91%, on day 2) with a median of 5 (IQR 3-7 on day 1 and IQR 3-8 on day 2) toileting events recorded each day. Toileting most commonly occurred at home (85/140, 61%, on day 1 and 129/171, 75%, on day 2) due to a need to go (114/140, 66%, on day 1 and 153/171, 74%, on day 2). The most common reasons for delaying toileting were “work duties” (33/140, 21%, on day 1 and 21/171, 11%, on day 2) and “errands or traveling” (19/140, 12%, on day 1 and 19/171, 10%, on day 2). Response to at least 1 look-back notification was similarly high (41/44, 93%, on day 1 and 42/44, 95%, on day 2), with number of responses higher on average on day 2 compared with day 1 (mean on day 1=3.2, 95% CI 3.0-3.5; mean on day 2=4.3, 95% CI 3.9-4.7; *P*<.001). Median additional toileting events reported on the look-back survey were 1 (IQR 1-2) and 2 (IQR 1-2) on days 1 and 2, respectively. Overall concordance between self-reported location recording and GPS was 76% (188/247). Participants reported lower urge ratings when at home versus away when reporting real-time toileting (median rating 61, IQR 41-84 vs 72, IQR 56-98), and daily fluid intake showed a small to medium positive correlation with toileting frequency (day 1 *r*=0.3, day 2 *r*=0.24). Toileting frequency reported in “Where I Go” showed a small positive correlation with the frequency item from the International Consultation on Incontinence Questionnaire (*r*=0.31 with day 1 toileting frequency and *r*=0.21 with day 2 toileting frequency).

**Conclusions:**

“Where I Go” has potential to increase the understanding of factors that affect women’s toileting decisions and long-term bladder health. We anticipate its use as a data collection tool in population-based studies.

## Introduction

Little is known about how women make decisions in real-time around toileting for urination, and how those decisions result in moment-to-moment specific behaviors to manage bladder needs in real-world environments, the surrounding environment (eg, man-made structures and natural settings) and the sociocultural world (eg, work, play, school, family, and other social settings) in the context of physiological factors (eg, age, fluid intake, emotion, and discomfort). Women with lower urinary tract symptoms (LUTS) report more frequently engaging in toileting behaviors such as hovering to urinate, delayed toileting or holding, and straining during toileting [1-5]. However, whether these examples of toileting behaviors contribute to LUTS, represent adaptations to the presence of symptoms, or a complex interaction between environmental, sociocultural, and physiological factors is unclear.

Variance within and across women in influences leading to real-time decisions about toileting is difficult to measure. Validated questionnaires such as the “Toileting Behaviors: Women’s Elimination Behaviors Scale” [6] are limited by reliance on recalled behaviors and do not assess potential influences on toileting decisions (eg, bathroom safety and cleanliness, personal or work activities) and reasons for toileting (eg, no time to go later, having a bowel movement, wanting a break, or changing pad or tampon) that occur at the time of initiating or delaying toileting. Although bladder (or frequency or volume) diaries are considered to have higher validity than surveys, as they can capture toileting frequency and volume, urgency ratings, type and amount of fluids consumed, and urinary incontinence episodes as they occur, they still do not capture specific reasons for toileting or delaying toileting. They are also limited by participant burden (which can affect adherence), recall bias, and administrative costs.

Due to the difficulty in measuring real-world influences by diary, survey, or interview, scientific studies lack evidence for many hypothesized relationships between sociocultural and physical environment factors and onset or worsening of LUTS or deterioration of bladder health. To address this gap, the Prevention of Lower Urinary Tract Symptoms (PLUS) Research Consortium developed a smartphone app called “Where I Go.” The Consortium’s goal was to produce a sophisticated data collection tool to explore potential relationships between factors influencing toileting decisions and bladder health. Given the increasing ubiquity of smartphone use in the United States (as of 2021, 85% of people in the United States own a smartphone and 11% own another type of cell phone) [7], PLUS designed Where I Go to provide dense, accurate data about moment-to-moment toileting decisions and bladder management behaviors in the real world. This diary-like app was designed to limit recalled information and maximize end users’ interests in the use of the data collection tool. A key design element was location tracking at the time of the toileting event. The objective was to produce a simple, easy, and fun way for women to record episodes of “thinking about my bladder,” and “now I peed,” along with influencing contextual factors and accurate location, that is, “where” the event took place.

The first report on Where I Go described the app’s design, build process, and usability or acceptability testing, including extensive supplementary material: multiple video clips of the build process and composition of the finished app as shown by a complete set of screenshots. Initial testing demonstrated above-average usability as measured by the System Usability Scale (SUS) [8-11] and “good” functionality as measured by the Mobile Application Rating Scale [12]. Where I Go also demonstrated high appeal to users (eg, color choices, intuitiveness, and delight in the cartoon features) as documented via qualitative data obtained postuse (Miller et al [11]). The purpose of this second report is to (1) demonstrate that Where I Go can capture nuanced and granular data in real-time or via short look-back periods, and (2) provide an initial description of the variation in novel parameters collected within Where I Go to support readiness for use in large population-based studies.

## Methods

### Ethical Considerations

After institutional review board approval, we used a cross-sectional study design with sampling from regions surrounding 4 PLUS research centers (Washington University in St. Louis [201912088], Loyola University Medical Center [LU212615], University of Pennsylvania [protocol number 833718], and University of Alabama at Birmingham [IRB-300003879]) to maximize variability across key novel Where I Go variables. We purposely excluded the University of Michigan PLUS Center where the build and prior informal iterative usability testing was performed. Women were instructed to enter real-time data in the Where I Go app whenever they were thinking about their bladder or using the toilet over 2 days. In addition, ecological momentary assessment (EMA) data were collected 3 to 5 times per day to capture contextual factors around toileting including stress, mood, and fluid intake in addition to potentially missed toileting events. EMA data were collected via equally spaced prompts across participants’ usual waking hours as reported in the app set-up process at first log-in.

### Sample Selection and Size

As published in our first report [11], we estimated that a minimal sample size of 40 women would be needed to evaluate an a priori cut-point score of 74 (representing the 70th percentile of SUS scores or a grade of B-) [13] on the SUS. Community-dwelling women were recruited through flyers, advertisements in local community centers, contacting participants from previous studies, social media, and word-of-mouth. Women were eligible if they (1) were 18 years and older; (2) were fluent in written and spoken English; (3) were able to stand or walk independently without assistance by another person (use of cane or walker allowed); (4) owned a smartphone (Android or iOS) on which calls could be made; (5) had downloaded at least 1 app in the past 6 months; (6) were willing to respond to Where I Go prompts or texts and input data about toileting behaviors for 48 consecutive hours, during which they were required to leave their home at least once, but not to change time zones; and (7) agreed to 2 in-person visits at the study site. Women were excluded if they had a physical or mental condition that prevented them from completing written questionnaires and interactions with Where I Go; lived in an institutional setting (ie, skilled nursing, long-term care, or rehabilitation center); were pregnant or had a neurogenic or congenital bladder condition; and were unable to independently use the toilet.

After consent, each participant downloaded and installed Where I Go onto her smartphone, and then entered basic demographic information and typical “wake” time and pinned her home and any other regularly frequented locations. Formal data collection began with a check-in notification the morning following the download, which was scheduled automatically based on the inputted “wake time.” Starting with this “wake time,” each woman was instructed to continue using Where I Go for 48 consecutive hours. Participants were provided with a modest honorarium for their involvement.

### Measurement Instruments

Where I Go was built at the University of Michigan’s Center for Health Communications Research (UM invention registration OTT Ref No: 2019‐292) using the Cordova dual platform (The Apache Software Foundation; simultaneous build for use on mobile phones of either iOS or Android operating systems).

As reported in detail elsewhere [11], Where I Go provides each woman with an opportunity for (1) real-time reporting and (2) EMA reporting of the last few hours. Real-time reporting allows the woman to report planning for toileting throughout her day, The app starts with an option to tap the opening screen of “I’m thinking about my bladder.” Once tapped, these screens follow: “How strong is your urge to pee?” (slider scale 0‐100), “When are you planning to pee?” (response options “now” or “later”), and if later, “Why aren’t you going to pee now?” (response options list “don’t have to pee that bad yet,” “no place to pee,” “work duties,” etc). The screen then changes to an option for tapping “Now I peed.” Once tapped, these screens follow: “When did you pee” (response items right now, 10, 20, or 30+ min ago), “Where did you pee?” (with tailored locations options, the last one being “new location”), “How strong was your urge to pee?” (slider scale), “Why did you pee” (eg, “had to go,” “no time to go later,” “wanted a break,” and “I leaked” ), and “If you had to wait before you peed, why did you wait?” (“work duties,” “errands or traveling,” “long line for toilet,” “no delay,” “peed when I needed to,” etc). Response option of “other - fill in” was also offered when applicable. An “other” response offered the option for typing a comment into the fill-in box to indicate the reason for delaying toileting. At each of these interactions with the app, geospatial data were gathered automatically.

In addition to real-time reporting, Where I Go has built-in EMA, which the woman sees as pushed “Check-In” notifications. Push notifications for additional assessments offer users the opportunity to update information in the app if the recording of a toileting event was missed in real time. The screen series starts with automatic feedback to the woman about her prior real time reports, “These are the times you said you peed. Are there more you want to report?” and she can input additional toilet events and approximate time they occurred.

Within the EMA section of the app, additional screens with questions are “During this time period, I felt…” with slider scale response options anchored at each end with calm or stressed (0=calm to 10=stressed) and similarly not busy to busy.

EMA check-ins offered reporting during the look-back period of “Any pain with peeing,” and “Any leakage with peeing.” These screens were followed by “How much liquid did you drink?” with response options of selecting drink portion-size pictorials to add, and finally a screen “Did any of these drinks have these ingredients?” with response options “caffeine,” “artificial sweeteners,” “alcohol,” and “none of these.” Push notifications offered women the opportunity to record information on variables that are easier or more accurately reported through short look-back periods over the interval of time since the last notification rather than in real time. Geospatial data were also gathered automatically at each one of these EMAs.

The International Consultation on Incontinence Questionnaire (ICIQ) Overactive Bladder Module [14], a validated measure assessing overactive bladder and its impact on quality of life, was administered to participants at the end of the study. Responses to novel measures collected by Where I Go were compared with responses to these validated survey items on frequency, urgency, and incontinence.

### Response Rate to Prompts on Key Factors of Interest

Given the novel nature of the app and the exploratory nature of this report, there were no a priori rules for evaluating participant response rate to prompts. To assess the accuracy of the location-pinning function (home or away from home), we compared participants’ GPS longitude and latitude pinned location of “home,” set at baseline, to the longitude and latitude automatically captured when a participant interacted with the app to record a real-time toileting event at home. For participants with a missing or inaccurate home location pinned at baseline, their home location was replaced with the longitude and latitude of their first toileting event when they indicated that they were at home.

### Statistical Analysis

To illustrate if plausible values were captured across the wide variety of types of data collected by Where I Go, descriptive statistics were calculated for the daily number of toileting events, average urinary urge ratings, number of locations reported, locations of each toileting event, and reasons for delaying toileting. In addition, descriptive statistics were calculated on the EMA component of the app (check-ins), comparing a number of locations, stress ratings, busy ratings, leakage episodes, toileting locations, fluid intake volume and ingredients, and management strategies across day 1 and day 2.

Descriptive statistics were calculated to portray the frequency or lack of use of real-time and EMA survey elements. The ability to record toileting events in real time was assessed by comparing real-time recordings to the number of missed toileting events documented in the EMA section. We evaluated beverage intake response rates to prompts as at least 1 fluid intake recorded in a day, making the logical assumption that all women would have consumed at least a minimal amount of fluid on each day. Finally, the relationships between quantities measured within the app and the ICIQ items were assessed.

Descriptive statistics were calculated separately for day 1 and day 2 and reported as frequency and percentage for categorical variables and median and IQR for continuous variables. For comparisons of continuous variables (eg, daily toileting frequency and daily intake volume), Pearson correlations were calculated. For comparisons of a continuous variable across levels of categorical variables (eg, urge ratings at home vs away), box plots were used for qualitative visual assessment. All analyses were completed in the SAS Enterprise Guide (version 9.3; SAS Institute).

## Results

### Participant Characteristics

A total of 44 women completed the baseline survey with 48 hours of interaction with Where I Go. Participants had a mean age of 44 (range 21‐85) years, with 25/44 (56%) of participants identifying as White, 30/44 (68%) working one or more jobs, and 32/44 (73%) having a college degree or higher. Most participants had 2 or 3 toilets in the home (32/44, 73%), and most used an Apple phone (33/44, 75%) to interact with the app. Additional details on demographic information are provided in Table 1.

**Table 1.** Summary of demographic information collected directly within the Where I Go app in a pilot study of community participants (N=44).

Characteristic	Values
Age (years), mean (range)	44.3 (21-85)
Age (years), n (%)
18‐25	8 (18.2)
26‐45	16 (36.4)
46‐65	15 (34.1)
≥66	5 (11.4)
Employment (check all that apply), n (%)	
Homemaker	5 (11.4)
Student	8 (18.2)
Not working or unable to work	8 (18.2)
Working one or more jobs	30 (68.2)
Income (US $), n (%)	
<25,000	9 (20.5)
25,000-49,999	9 (20.5)
50,000-99,999	15 (34.1)
≥100,000	9 (20.5)
Missing	2 (4.5)
Sought care from health care provider for bladder, n (%)	
Yes	9 (20.5)
Missing	1 (2.3)
Education, n (%)	
High school diploma, General Educational Development Test, or less	0 (0)
Some college	12 (27.3)
Associate or Bachelor degree	18 (40.9)
Master degree	11 (25)
Professional or Doctorate degree	3 (6.8)
Hispanic or Latino, n (%)	8 (18.2)
Primary language spoken at home (check all that apply), n (%)	
English	43 (97.7)
Spanish	3 (6.8)
Other	2 (4.5)
Race (check all that apply), n (%)	
American Indian or Alaska Native	1 (2.3)
Asian	4 (9.1)
Black or African-American	12 (27.3)
Middle Eastern or North African	1 (2.3)
Native Hawaiian or other Pacific Islander	0 (0)
Other race or ethnicity or origin	1 (2.3)
White or Caucasian	25 (56.8)
Participant did not answer	3 (6.8)
Number of toilets in participants’ home?	
1	12 (27.3)
2	23 (52.3)
3	9 (20.5)
Phone type	
Android	10 (22.7)
Apple	33 (75)
Missing	1 (2.3)

### Real-Time Assessment of Toileting

On both days 1 and 2, the median number of toileting events recorded in real time was 5 (IQR 3‐7 on day 1 and IQR 3‐8 on day 2) and most toileting events were recorded at the time of the event (112/140, 80%, on day 1 and 136/171, 80%, on day 2, Table 2). The most common toileting locations were home (85/140, 61%, on day 1 and 129/171, 75%, on day 2) and workplace (34/140, 24%, on day 1 and 25/171, 15%, on day 2). Most participants reported toileting due to a need to go (114/140, 66%, on day 1 and 153/171, 74%, on day 2), with work duties and errands or traveling as common reasons for delaying toileting as well as selection of “other” both when reporting toileting now and later (Table 2).

Real-time urge ratings were similar across day 1 (median rating 64, IQR 52‐81) and day 2 (median rating 62, IQR 46‐85) when participants reported toileting now (Table 2). Ratings were lower but similarly consistent across days when participants reported toileting later (day 1 median 54.5, IQR 34-74; day 2 median 55, IQR 38-71).

**Table 2.** Real-time assessment descriptive statistics.

Construct	Day 1	Day 2
Number of voids^a^, median (IQR)	5 (3-7)	5 (3-8)
Number of locations, median (IQR)	1 (1-2)	2 (1-2)
Urge rating^b^, median (IQR)	60.5 (44.5-79.5)	60 (41-81)
When planning to pee		
Now, n (%)	140 (66)	171 (69)
Urge rating^b^, median (IQR)	64 (52-81)	62 (46-85)
When did you pee, n (%)		
Right now	112 (80)	136 (80)
10 minutes ago	11 (8)	20 (12)
20 minutes ago	2 (1)	5 (3)
30+ minutes ago	15 (11)	10 (6)
Where did you pee, n (%)		
Home	85 (61)	129 (75)
Workplace	34 (24)	25 (15)
School	6 (4)	1 (1)
Restaurant	1 (1)	8 (5)
Gym	5 (4)	2 (1)
Place of worship	1 (1)	1 (1)
Retail store	2 (1)	1 (1)
Gas station	1 (1)	0 (0)
Prefer not to say	0 (0)	0 (0)
Other	5 (4)	4 (2)
Why did you pee^c^, n (%)		
Had to go	114 (66)	153 (74)
No time to go later	10 (6)	12 (6)
My usual time to go	14 (8)	10 (5)
Went with others	0 (0)	0 (0)
Bowel movement	14 (8)	19 (9)
Wanted a break	4 (2)	2 (1)
Changed pad or tampon	5 (3)	5 (2)
I leaked	0 (0)	3 (1)
Other	13 (7)	4 (2)
Delay reason^c^, n (%)		
No delay. Peed when I needed	77 (50)	108 (59)
Work duties	33 (21)	21 (11)
Errands or traveling	19 (12)	19 (10)
Caring for family	9 (6)	10 (5)
Dirty bathroom	2 (1)	1 (1)
Bathroom not private or safe	0 (0)	1 (1)
Long line for toilet	4 (3)	3 (2)
Other	10 (6)	21 (11)
Later, n (%)	72 (34)	78 (31)
Urge rating^b^, median (IQR)	54.5 (34-74)	55.0 (38-71)
Delay reason^c^, n (%)		
Don’t have to pee that bad yet	11 (23)	7 (16)
No place to pee	6 (13)	5 (11)
Work duties	11 (23)	5 (11)
Errands or traveling	7 (15)	6 (14)
Caring for family	1 (2)	3 (7)
Dirty bathroom	0 (0)	1 (2)
Bathroom not private or safe	0 (0)	0 (0)
Long line for toilet	1 (2)	0 (0)
Other	10 (21)	17 (39)

a Median number of voids including real-time assessment and EMA survey was 6 (IQR 4-7) on day 1 and 6 (IQR 5-8) on day 2.

b Urge rating: 0=no urge, 100=strong urge.

c Multiple reasons per void possible.

### EMA Assessment of Toileting

A total of 6 out of 44 participants (14%) on day 1 reported toileting via the EMA survey only and did not use the real-time function. Similarly, on day 2, four out of 44 participants (9%) used the EMA survey only. In contrast, 3 participants out of 44 (7%) on day 1 and 2 participants out of 44 (5%) on day 2 did not respond to any EMA notifications. The average number of responses to the EMA survey was higher on day 2 than on day 1 (day 1=3.2, day 2=4.3; *P*<.001). On the EMA survey, participants reported a median of 1 (IQR 1‐2) additional toileting event on day 1 and 2 (IQR 1‐2) additional toileting events on day 2 (Table 3).

**Table 3.** Ecological momentary assessment (EMA) descriptive statistics.

Construct	Day 1	Day 2
Voiding-related		
Number of voids^a^ added, median (IQR)	1 (1-3)	2.5 (1-4)
Number of locations, median (IQR)	1 (1-2)	2 (1-2)
Pain while peeing, n (%)	1 (1)	1 (1)
Leakage episodes, median (IQR)	0 (0-0)	0 (0-0)
Where did you pee, n (%)		
Home	45 (56)	73 (68)
Workplace	28 (35)	15 (14)
School	1 (1)	1 (1)
Restaurant	2 (3)	3 (3)
Gym	0 (0)	1 (1)
Place of worship	1 (1)	1 (1)
Retail store	1 (1)	2 (2)
Gas station	0 (0)	2 (2)
Prefer not to say	0 (0)	1 (1)
Other	2 (3)	8 (8)
Intake-related		
Fluid intakes added, median (IQR)	3 (2-4)	4 (3-5)
Approximate daily intake volume (oz), median (IQR)	32 (20-40)	41 (20-60)
Intake ingredient^b^, n (%)		
Caffeine	38 (30)	45 (25)
Artificial sweeteners	8 (6)	15 (8)
Alcohol	1 (1)	5 (3)
None of these	78 (62)	115 (64)
Environment-related		
Stress rating^c^, median (IQR)	1 (0-3)	2 (1-3)
Busy rating^d^, median (IQR)	4 (1-7)	4 (1-6)
Highly focused, n (%)	103 (67)	130 (65)
Management strategies, n (%)		
Peed at certain times of the day because of schedule	32 (18)	21 (9)
Peed before certain activities	26 (15)	32 (14)
Knew I couldn’t go, so I wore a pad or diaper	0 (0)	1 (0.5)
Limited drinking	8 (5)	6 (3)
Had the urge but ignored it	11 (6)	17 (7)
No strategy; I just peed when I needed to	99 (55)	151 (66)
I used some other strategy	2 (1)	0 (0)

a Median number of voids including real-time assessment and EMA survey was 6 (IQR 4-7) on day 1 and 6 (IQR 5-8) on day 2.

b Drink-level, multiple ingredients per participants per day possible; multiple ingredients per intake possible.

c Stressing rating: 0=calm, 10=stress.

d Busy rating: 0=not busy, 10=busy.

### Location Assessment

A total of 15 out of 44 participants (34%) had a missing or incorrect pinned home location at baseline. Two participants out of 44 (5%) did not have a pinned home location at baseline and 13 out of 44 (30%) had a pinned home location that was set incorrectly. Of all toileting events that had location automatically pinned by the app as “Home,” 97% (103/106) of those events were self-reported as having occurred at home. However, for toileting events where participants’ location was automatically pinned as “Away,” only 60% (85/141) were self-reported by those participants as being away from home, potentially due to an incorrectly pinned home location.

### Look-Back Factors Potentially Influencing Toileting Decisions

Median number of beverage servings put into the EMA beverage section ranged from 3 to 4 per day, and the approximate median daily intake volume was 32 (IQR20-40) to 41 (IQR 20-60) ounces. While most participants did not report using strategies to manage their toileting events, those that did most reported toileting at certain times of the day due to their schedule (32/178, 18%, on day 1 and 21/228, 9%, on day 2) and before certain activities (26/178, 15%, on day 1 and 32/228, 14%, on day 2). Most participants reported low stress generally (median scores 1‐2 on a scale from 0=calm to 10=stressed) during the look-back periods.

### Within App Comparisons

**Figure 1.**
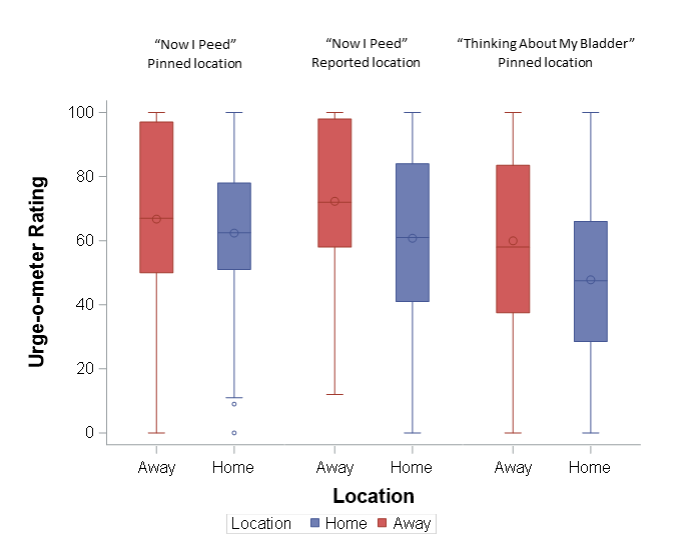
Comparisons between urge-o-meter ratings at home versus away from “Now I Peed” and “Thinking About my Bladder” surveys (The center line of the boxplot represents the median, while the center circle represents the mean. The outer edges of the boxplot represent the 25th-75th percentiles. Whiskers extend to 1.5 times the IQR. Outliers [values with distance>1.5*IQR] are represented by circles outside of the whiskers).

**Figure 2.**
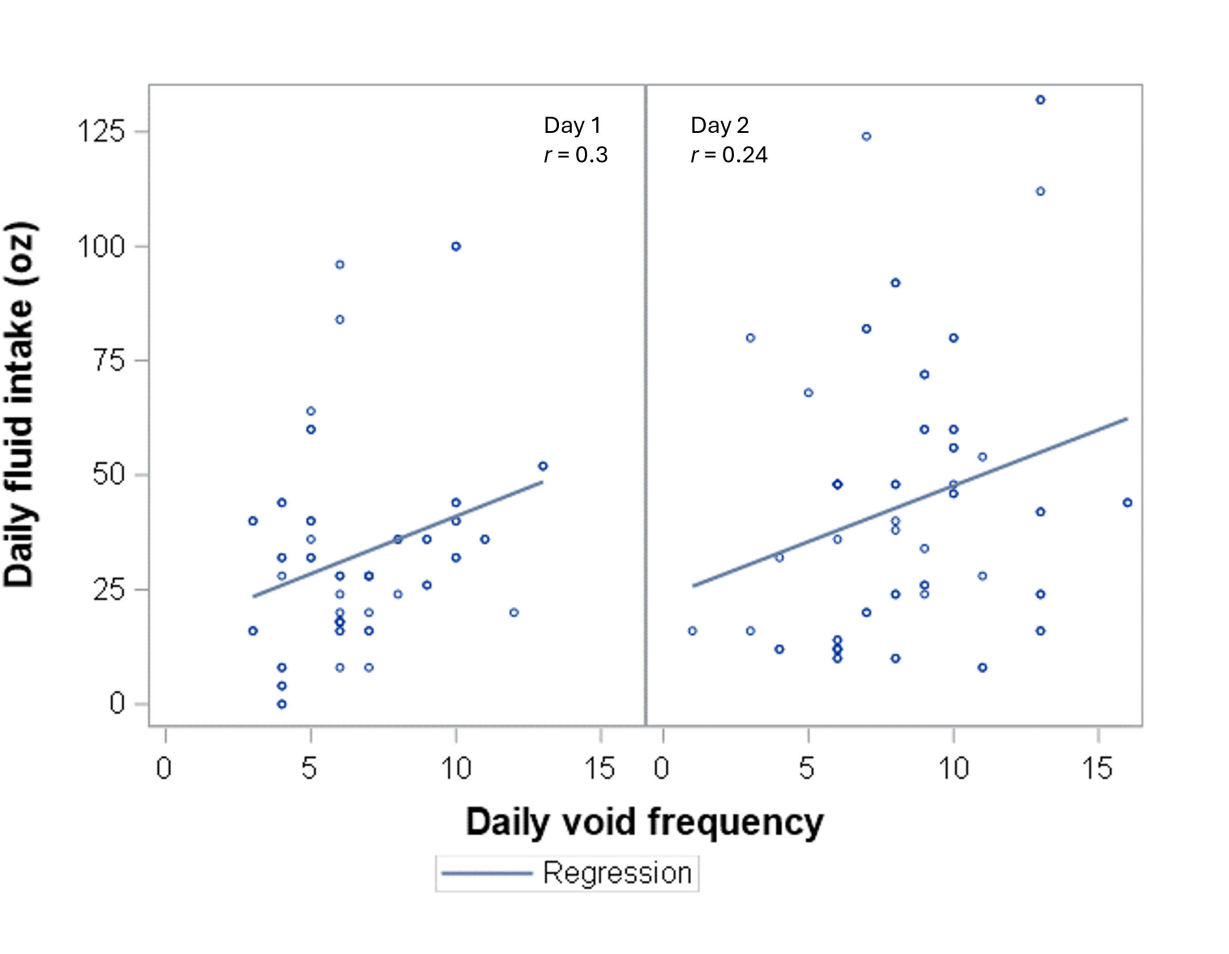
Comparison between daily fluid intake and daily void frequency.

**Figure 3.**
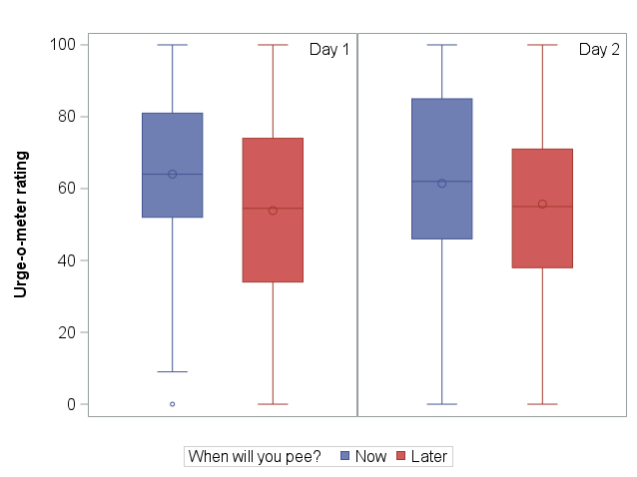
Distribution of urge-o-meter ratings: pee now versus pee later (The center line of the boxplot represents the median, while the center circle represents the mean. The outer edges of the boxplot represent the 25th-75th percentiles. Whiskers represent data points outside the intra-quartile range but are not considered outliers. Outliers [values>1.5*IQR] are represented by circles outside of the whiskers).

When comparing urge ratings between “Home” and “Away,” urge ratings tended to be lower on average when participants were at home (Figure 1). This relationship was more pronounced for the “Now I Peed” survey using participants’ reported location (median rating 72, IQR 56-98, when away vs 61, IQR 41-84, when at home) and the “Thinking About My Bladder” survey using participants’ pinned location (median rating 58, IQR 41.5-74.5, when away vs 48, IQR 24-67, when at home). Daily fluid intake had a small to medium positive correlation with daily toileting frequency (day 1 *r*=0.3, day 2 *r*=0.24, Figure 2). In addition, participants typically reported higher urge ratings when they reported they would “Pee Now” versus “Pee Later,” (median 64, IQR 52-81 vs 54.5, IQR 34-74, on day 1; median 62, IQR 46-85 vs 55, IQR 38-71, on day 2; Figure 3).

### Relationships With ICIQ Responses

**Figure 4.**
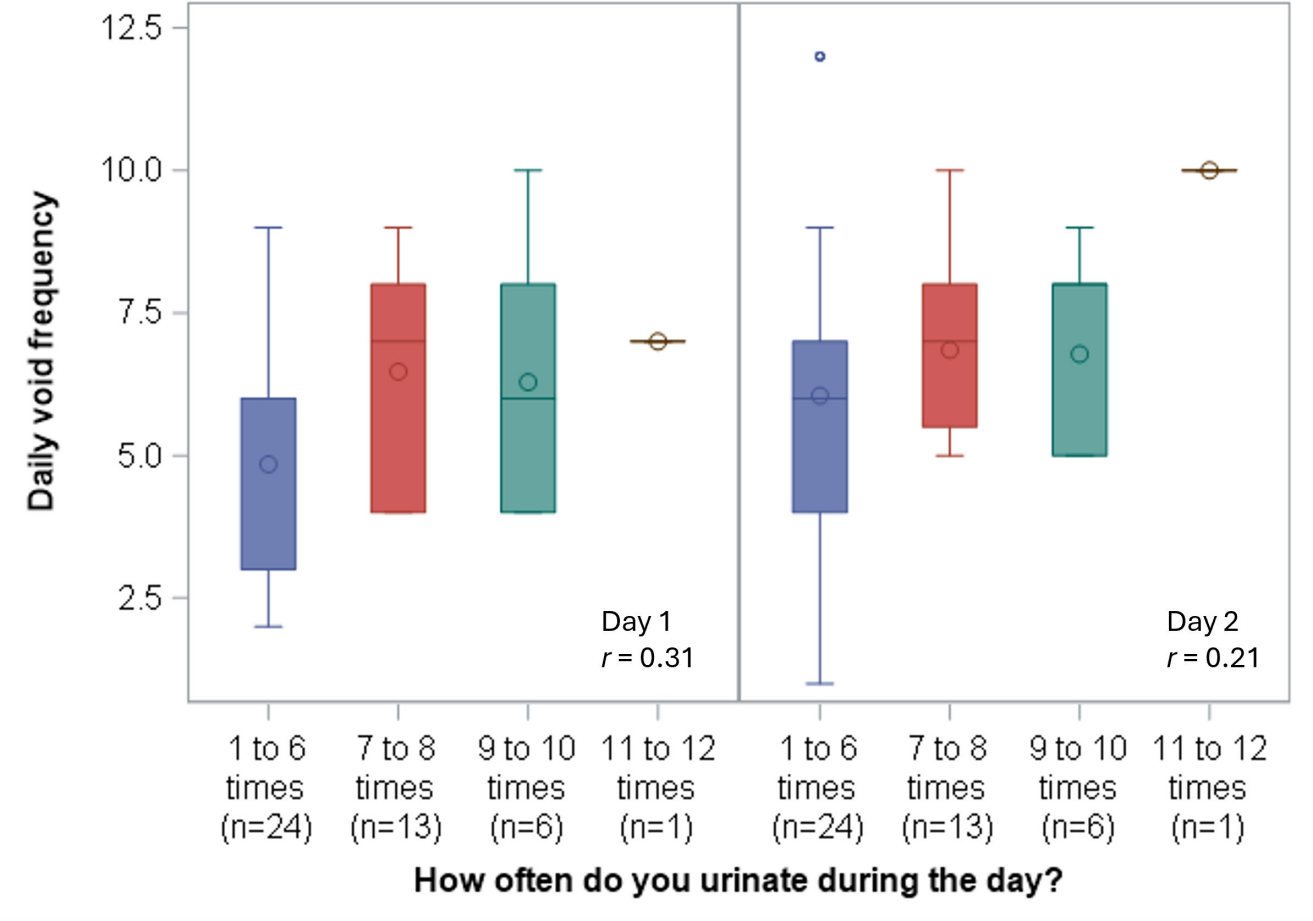
Distribution of daily voids (app) by International Consultation on Incontinence Questionnaire frequency category. (The center line of the boxplot represents the median, while the center circle represents the mean. The outer edges of the boxplot represent the 25th-75th percentiles. Whiskers represent data points outside the intra-quartile range but are not considered outliers. Outliers [values>1.5*IQR] are represented by circles outside of the whiskers).

Participants’ daily toileting frequency reported in Where I Go showed a small positive correlation with the daily urinary frequency item on the ICIQ (*r*=0.31 with day 1 toileting frequency and *r*=0.21 with day 2 toileting frequency, Figure 4). Most participants reported urination frequencies of 1 to 6 times and 7 or 8 times on the ICIQ frequency item (37/44, 84%); therefore, the relationship between toileting events recorded in Where I Go and ICIQ responses cannot be assessed precisely for those reporting urination frequency greater than 8 times per day on the ICIQ.

## Discussion

In this paper, we report on the 2-day experience of 44 community women with the Where I Go app. Toileting behaviors, decisions around toileting, toileting location, and beverage consumption were recorded using real-time input by the user, EMA, and geospatial data [11]. This data collection tool’s outputs, as described above, illustrate the wide range of data that Where I Go collects and the resulting plausible values, relationships, and preliminary variability of those parameters. The agreement with an extant survey instrument in the field is another positive indicator of its value. Strengths of this data collection tool include its continuous surveillance, user prompts, dynamic visualizations (eg, facial expressions to help users’ rate their level of urinary urge before toileting), ecofriendly nature relative to paper diaries, and convenience.

Where I Go uses both real-time and EMA functions with high compliance, minimizing recall concerns typically associated with surveys or questionnaires. In addition, Where I Go has the capacity to capture contextual parameters and adaptive behaviors such as limiting fluid intake, toileting on a schedule, preemptive toileting, and use of absorbent products, all of which are missing from current tools. These findings support the utility of Where I Go for conducting large-scale population research. Users must own a smartphone and be able to download a mobile app that is designed to be used on either Android or iOS smartphones. The ability to measure location, as demonstrated by Where I Go, is a key advantage over paper diaries. The agreement between participant self-report and pinned GPS location was high overall (188/247, 76%). However, despite the seeming ubiquity of smartphones, this requirement may limit the inclusion of certain populations, particularly marginalized and minoritized populations, who may have more limited access to both smartphones as well as the data required to operate the app.

Where I Go offers opportunities to explore the impact of social and environmental determinants of toileting behavior from the individual to the societal level. Reasons for delaying voiding captured by Where I Go could reveal underlying factors impacting LUTS, from personal or work responsibilities, issues of privacy, cleanliness, and safety, to the lack of access to public toilets [15-18]. Delaying urination or holding urine to the point of discomfort or urgency may be a risk factor for bladder symptoms. Prior work has shown that women who limited their use of workplace toilets had higher prevalences of urinary urgency, overactive bladder symptoms, and urinary leakage, which could be more robustly investigated through large-scale use of Where I Go [19].

This study allowed us to identify areas of future improvement in Where I Go. The most notable future improvement expected is in the initial home location pinning. Discordance between app-recorded and self-reported location was most common when participants reported toileting events at home, but the app GPS did not match their home location. Whether this was due to technological issues (eg, poor service at a participants’ home location) or user error is unknown and should be investigated further. The ability of smartphone GPS systems to identify precise locations is improving over time, however, which should positively impact the utility of Where I Go. Other improvements include the exploration of reasons typed into the app when participants selected “other” with the goal of honing the list of given response options. Currently, Where I Go does have an automatic function that brings in the “other” option’s typed-in response options when the participant is next queried. Anecdotally, women appreciated this feature.

Results from this study support the implementation of Where I Go as an important data collection tool for population-based studies investigating factors affecting bladder health; however, the small sample did not allow us to demonstrate its use in all subgroups, including individuals with lower education and higher income. Future work should include the use of this app among a more diverse and larger population, with adequate power for testing hypothesized relationships. In addition, data collection occurred over a 2-day period; however, the optimal data collection time frame is still to be determined. The app as currently built does offer the ability to extend data collection beyond 2 days. Finally, average daily fluid intake was lower than expected, potentially due to underreporting.

More research should also be directed towards comparing data obtained by Where I Go with validated patient-reported outcome measures (PROMs) on bladder health and LUTS, with an emphasis on environmental factors that may limit toileting [20]. When comparing traditional bladder diaries to PROMs, other studies have found that bladder diary data are comparable to PROMs for women with LUTS [20]. However, this small sample with low levels of LUTS demonstrated only weak associations with items from the ICIQ.

Where I Go measures toileting and associated symptoms across a fluidity of environments. The breadth of environments in a woman’s day of managing her bladder function is not commonly measured in traditional paper or electronic diaries. Real-time data recording supplemented by EMA opportunities each day overcomes recall concerns inherent in most extant survey instruments and offers more nuanced data than standard surveys or bladder diaries.

We conclude that the “Where I Go” app is an instrument that can help us understand a fuller spectrum of potential factors that affect women’s bladder health. The results reported support this novel data collection tool’s use in a wider study; specifically in a general population of women with and without LUTS.

## Abbreviations

CHCR: Center for Health Communications Research
EMA: ecological momentary assessment
ICIQ: International Consultation on Incontinence Questionnaire
LUTS: lower urinary tract symptoms
PLUS: Prevention of Lower Urinary Tract Symptoms
PROM: patient-reported outcome measure
SUS: System Usability Scale

## References

[1] Willis-Gray MG, Wu JM, Sripad A, Newman D (2017). Toileting behaviors in women presenting to a urogynecology clinic. UNJ.

[2] Palmer B, Kropp B (2018). Urologic evaluation and management of pediatric kidney transplant patients. Urol Clin North Am.

[3] Newman DK, Burgio KL, Cain C (2021). Toileting behaviors and lower urinary tract symptoms: a cross-sectional study of diverse women in the United States. Int J Nurs Stud Adv.

[4] Kowalik CR, Casteleijn FM, van Eijndhoven HWF, Zwolsman SE, Roovers JPWR (2018). Results of an innovative bulking agent in patients with stress urinary incontinence who are not optimal candidates for mid-urethral sling surgery. Neurourol Urodyn.

[5] Daily AM, Kowalik CG, Delpe SD, Kaufman MR, Dmochowski RR, Reynolds WS (2019). Women with overactive bladder exhibit more unhealthy toileting behaviors: a cross-sectional study. Urology.

[6] Wang K, Palmer MH (2011). Development and validation of an instrument to assess women’s toileting behavior related to urinary elimination: preliminary results. Nurs Res.

[7] Mobile fact sheet. Pew Research Center.

[8] Brooke J (1986). System usability scale (SUS): a quick-and-dirty method of system evaluation user information.

[9] Bangor A, Kortum PT, Miller JT (2008). An empirical evaluation of the System Usability Scale. Int J Hum Comput Interact.

[10] Bangor A, Kortum P, Miller J (2009). Determining what individual SUS scores mean: adding an adjective rating scale. J Usability Stud.

[11] Miller J, Wyman J, An L (2024). Design of a tool capable of assessing environmental sociocultural physical factors influencing women’s decisions on when and where to toilet within real-world settings: protocol for the build and usability testing of a mobile app for use by community-dwelling women. JMIR Res Protoc.

[12] Stoyanov SR, Hides L, Kavanagh DJ, Zelenko O, Tjondronegoro D, Mani M (2015). Mobile app rating scale: a new tool for assessing the quality of health mobile apps. JMIR mHealth uHealth.

[13] Sauro J (2011). A Practical Guide to the System Usability Scale: Background, Benchmarks & Best Practices.

[14] Jackson S, Donovan J, Brookes S, Eckford S, Swithinbank L, Abrams P (1996). The Bristol female lower urinary tract symptoms questionnaire: development and psychometric testing. Br J Urol.

[15] Yuko E Where did all the public bathrooms go?. Bloomberg.

[16] Smoyer AB, Pittman A, Borzillo P (2023). Humans peeing: justice-involved women’s access to toilets in public spaces. PLoS One.

[17] Maroko AR, Hopper K, Gruer C, Jaffe M, Zhen E, Sommer M (2021). Public restrooms, periods, and people experiencing homelessness: an assessment of public toilets in high needs areas of Manhattan, New York. PLoS One.

[18] Sommer M, Gruer C, Smith RC, Maroko A (2020). Menstruation and homelessness: challenges faced living in shelters and on the street in New York City. H Place.

[19] Reynolds WS, Kowalik C, Delpe SD, Kaufman M, Fowke JH, Dmochowski R (2019). Toileting behaviors and bladder symptoms in women who limit restroom use at work: a cross-sectional study. J Urol.

[20] Flynn KE, Wiseman JB, Helmuth ME (2022). Comparing clinical bladder diaries and recalled patient reports for measuring lower urinary tract symptoms in the symptoms of Lower Urinary Tract Dysfunction Research Network (LURN). Neurourol Urodyn.

